# Neural Aspects of Prospective Control through Resonating Taus in an Interceptive Timing Task

**DOI:** 10.3390/brainsci12121737

**Published:** 2022-12-19

**Authors:** F. R. (Ruud) van der Weel, Ingemārs Sokolovskis, Vicente Raja, Audrey L. H. van der Meer

**Affiliations:** 1 Developmental Neuroscience Laboratory, Department of Psychology, Norwegian University of Science and Technology (NTNU), 7491 Trondheim, Norway; 2 Kavli Institute for Systems Neuroscience, Norwegian University of Science and Technology (NTNU), 7491 Trondheim, Norway; 3 Department of Philosophy, University of Murcia, 30100 Murcia, Spain; 4 Rotman Institute of Philosophy, Western University, London, ON N6A 5B7, Canada

**Keywords:** interceptive timing, tau-coupling, prospective control, HD EEG, resonance, ecological neuroscience

## Abstract

High-density electroencephalography from visual and motor cortices in addition to kinematic hand and target movement recordings were used to investigate τ-coupling between brain activity patterns and physical movements in an interceptive timing task. Twelve adult participants were presented with a target car moving towards a destination at three constant accelerations, and an effector dot was available to intercept the car at the destination with a swift movement of the finger. A τ-coupling analysis was used to investigate involvement of perception and action variables at both the ecological scale of behavior and neural scale. By introducing the concept of resonance, the underlying dynamics of interceptive actions were investigated. A variety of one- and two-scale τ-coupling analyses showed significant differences in distinguishing between slow, medium, and fast target speed when car motion and finger movement, VEP and MRP brain activity, VEP and car motion, and MRP and finger movement were involved. These results suggested that the temporal structure present at the ecological scale is reflected at the neural scale. The results further showed a strong effect of target speed, indicating that τ-coupling constants *k* and *k_res_* increased with higher speeds of the moving target. It was concluded that τ-coupling can be considered a valuable tool when combining different types of variables at both the ecological and neural levels of analysis.

## 1. Introduction

It is essential for survival that humans guide the movements of their hands and feet to objects and places on surfaces, as when grasping things or securing footing when running. The guidance must be prospective. It requires perceptual information about what is going to happen next, allowing the ongoing movement to be extrapolated so that timely adjustments to the movement can be made. In general, guiding an effector so that it makes contact with a surface or object at the right place, at the right time, and with the right force or velocity of impact requires simultaneously regulating the rates of closure of several gaps: the spatial gap between the effector and surface or object, the spatial gap between the effector and the destination in the environment where interception is to take place, and the gap between the current body posture and the posture to be reached as contact is made [1].

Extensive research has studied human behavior and the pick-up of action-relevant perceptual information during interception tasks [2,3,4,5,6,7], the timing of movement initiation [8,9,10,11], and the sensory guidance of ongoing movement [12,13,14,15,16,17,18,19,20,21], yet little is known about the relation between the pick-up of predictive perceptual information during interceptive actions and the neural mechanisms that accompany it.

**Figure 1:**
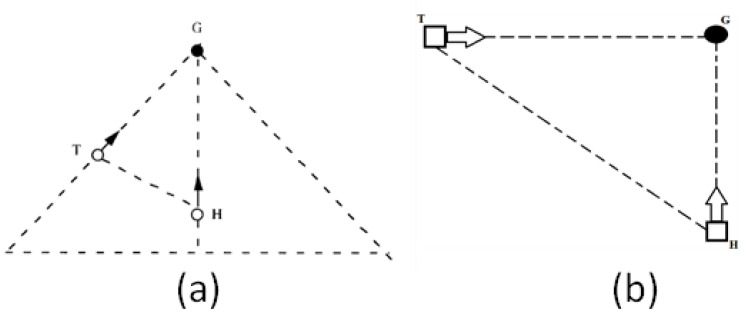
(**a**) Diagram of the experimental task used in [1] that inspired (**b**) the present experiment which required the subject to move the hand (H) on a large vertical touchscreen monitor to collide with a horizontally moving target (T) in goal location G just as the moving target reached G. Note that the interception task in [1] involved moving a computer mouse whereas the present task involved whole arm movement.

The aim of the present paper was to explore the relation between perception, movement, and brain activity during prospective action in an interceptive timing task. We investigated perceptual guidance of contact together with its accompanying brain activity where the hand had to be moved to intercept a moving target just as it passed through a particular location. In the experiment, the target (T) moved along a horizontal path at various constant accelerations and the hand (H) was moved vertically up the screen to intercept T when it reached the goal location G (Figure 1).

### 1.1. Tau-Coupling

We tested the hypothesis that guidance of contact is achieved by τ-coupling. τ-coupling is an aspect of general τ (tau) theory. According to Lee and colleagues [1,22], general τ theory proposes that a central task in guiding movement is controlling the closure of physical gaps between effectors and their goals—e.g., distance gaps as when reaching, angular gaps as when redirecting gaze, and so on. General τ theory hypothesizes that online guidance of gap closure can be achieved by continuously sensing a single variable, namely the time-to-closure of the gap at the current closure rate. This is referred to as τ of the gap. Perceiving the changing size of the gap or its rate of closure is not necessary as the τs of physical gaps can, in principle, be directly sensed through the τs of corresponding sensory gaps between elements in the input arrays. One means for guiding movement, including closing two physical gaps synchronously, could be by τ-coupling the τs of gaps, i.e., keeping the τs in constant ratio. Evidence supporting the idea of τ-coupling is reviewed in [16].

To illustrate the idea of τ-coupling, consider the two gaps, HG and TG (Figure 1). To succeed in the task, these gaps must close simultaneously. Although the gaps will generally differ in size and closing speed during the movement, they could be closed simultaneously simply by keeping the τs of the gaps, τ_HG_ and τ_TG_, τ-coupled in constant ratio, such that

τ_HG_ = *k*τ_TG_ (1)

for a constant *k*. This is because, as gap TG closes, τ_TG_ becomes zero. Additionally, if Equation (1) holds, τ_HG_ and gap HG become zero at the same time. τ-coupling is, therefore, a procedure to solve the experimental task. Additionally, it is known that the parameter *k* regulates aspects of the terminal kinematics of closure of the HG gap [23]. For 0 < *k* < 1, this would result in H arriving at the goal location G simultaneously with T and stopping there. If 0 < *k* < 0.5, the closure rate of gap HG will get steadily lower at a decreasing rate and will reach zero as the gap closes (i.e., soft contact). Finally, if 0.5 < *k* < 1, the closure rate of gap HG will again get steadily lower but now at an increasing rate until that rate reaches a maximum, with the result that the final closure rate will be positive as the gap closes (i.e., hard contact). In general, the higher the value of *k* greater than 0.5, the steeper the decrease in closure rate and the higher the closure rate at contact. If *k* > 1, the closure rate of the gap will not decrease at all but will steadily increase towards and past the goal location G, resulting in accelerating contact. In the current experiment, the target car (T) moved along a horizontal path at three different constant accelerations. As a result, resulting *k* values in a τ-coupling between the two gaps between HG and TG are expected to reflect the constant acceleration approach and will consequently lie around 1.

Evidence for τ-coupling on an interception task similar to the present experiment has been found by [1]. However, what about the involvement of the nervous system in this process? Adding to the promising attempts to include the nervous system using general tau theory [24,25], the aim of the next section is to extend theoretical control principles of τ-coupling to also include the functioning of the nervous system. To achieve this, we will first introduce the more general concept of resonance.

### 1.2. Resonance

Resonance occurs when a system A drives a system B to oscillate at a greater amplitude at one or more frequencies [26,27]. An example of resonance is observed when a person pushes a swing. In this situation, the amplitude of the swinging increases when the swing is rhythmically pushed at appropriate moments—i.e., moments related to the resonant frequency of the swing. The pushing goes against the swing’s motion when performed at other moments of the movement cycle. Another example is observed when the whole guitar body resonates to vibrating strings while being played. Of course, guitars resonate to their playing in very complex ways (e.g., involving harmonics), but the basic physical principle of resonance remains the same.

Resonance appears in experimental neuroscience related to topics such as single neuron activity [28], learning through resonance frequency shifts in subthreshold oscillations in the brain [29,30,31], or mirror neurons [32]. It is also present in computational neuroscience, where resonance takes different forms in the study of artificial neural networks: stochastic resonance [33], coherence resonance [34], network resonance [35], or adaptive resonance theory [36,37], among others. The field of robotics and artificial intelligence has made use of the notion of resonance as well [38], and, of course, it has been used in the tradition of ecological psychology [26,27,39,40,41,42], which is the most relevant one for this study.

**Figure 2:**
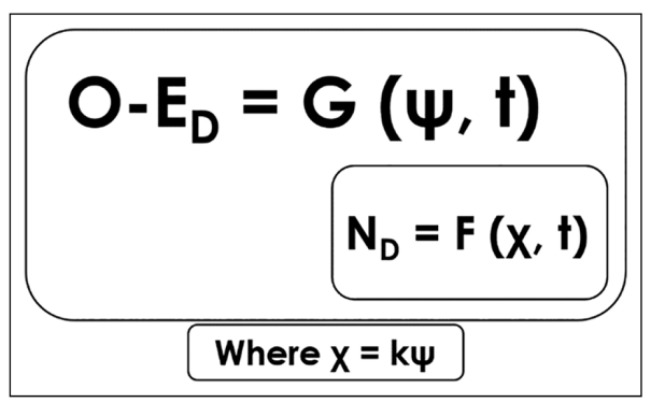
Model of resonance. O-E_D_ is characterized as a function G of ecological information ψ and time. N_D_ is characterized as a function of the variable χ and time. Ecological resonance occurs when ψ = *k_res_*χ, where *k* = *k_res_*. Reproduced from [27] (p. 409).

The ecological notion of resonance, as operationalized by Raja [26,27,41], see also [43], provides a characterization of the mechanism that allows perceptual systems to detect ecological information. According to ecological psychologists, organisms find information in the ambient energy arrays that surround them—e.g., in the surrounding light, in the surrounding air, in the surrounding chemicals, etc. Information has to do with the structure of those arrays as they interact with the layout of the environment. For instance, the structure of light in each place depends on the position of the source of light and the surfaces off which it bounces. Therefore, the ambient *optic* array has particular structures in different places and with different light conditions. These structures are the ecological information which the organisms’ perceptual systems resonate to. More formally, ecological information is a variable that features in the dynamical model of the organism–environment interactions and that serves as a constraint for such interactions (see Figure 2). Ecological resonance occurs when the same variable of ecological information that features in the dynamical models of these interactions also constrains brain dynamics. In other words, when the dynamics of brain activity are constrained by the same variable of ecological information that constrains the organism-environment dynamics, the situation is an instance of ecological resonance. For instance, in a situation in which the variable of ecological information τ constrains a given organism–environment interaction, ecological resonance occurs when that very variable τ is also found to constrain the organism’s brain dynamics in the situation (see [42]). This is also an instance of what we would call τ-coupling at two different scales, the organism–environment one and the neural one.

The general formalism of ecological resonance for which τ-coupling would be a particular example is predicated on the dynamics of a neural system (N_D_) nested within the dynamics of an organism–environment system (O-E_D_). This is, of course, the usual case of brains within bodies within environments. Then, ecological resonance occurs when whatever variable ψ of ecological information constraining O-E_D_ is proportional to a variable χ constraining N_D_, such that ψ = *k_res_*χ, where *k_res_* is the parameter of resonance (Figure 2). In the concrete case of our study, when the interaction at the O-E_D_ is described by using the variable tau (τ), the interaction at the N_D_ scale must be explained by appealing to the same variable τ. In this sense, the parameter *k* in Equation (1) will be effectively capturing an instance of ecological resonance and, therefore, *k* = *k_res_*.

The biological plausibility of ecological resonance in the form of using τ-coupling has already been shown [42]. The present study furthers this work by investigating the interceptive task described in Figure 1b. We studied five different τ-coupling events that are thought to best describe activity at the ecological scale and the neural scale. These are the τ-couplings of: (1) the target motion towards the goal location (TG) with time-to-collision, (2) the target motion towards the goal location (TG) with hand movement towards the goal location (HG), (3) the visual cortex activity (VEP, visual evoked potential) with motor cortex activity (MRP, motor-related potential), (4) the visual cortex activity (VEP) with target motion (TG), and (5) the motor cortex activity (MRP) with hand movement (HG).

To solve the interceptive timing task, participants were expected to use a τ-coupling strategy to intercept moving target T, where time-to-closure of the action gaps between HG and TG would be kept constant. Further, couplings between perception, motor, and brain activity were measured and explored in detail. It was expected that when analyzing the temporal dynamics of perceptuomotor behavior HG and its accompanying neural activity, empirical evidence can be provided that the temporal structure of different interceptive actions is sustained during the neural control of these actions.

## 2. Materials and Methods

### 2.1. Participants

A convenience sample consisting of seventeen young adults, aged between 21 and 27 (mean age 24), were recruited for this study at the Norwegian University of Science and Technology (NTNU) in Trondheim. From these, twelve participants (six females) provided datasets of good quality and relatively free from artefacts for further brain analyses and were included in the study. All participants reported that they were right-handed.

Electroencephalography (EEG) is a non-invasive technique and causes no known physical harm to participants. Before the experiment started, participants gave their written consent and were informed about their rights to stop the experiment or withdraw from the study at any given time. The local Regional Ethics Committee and the Norwegian Centre for Research Data approved the study.

### 2.2. Experimental Stimuli and Paradigm

**Figure 3:**
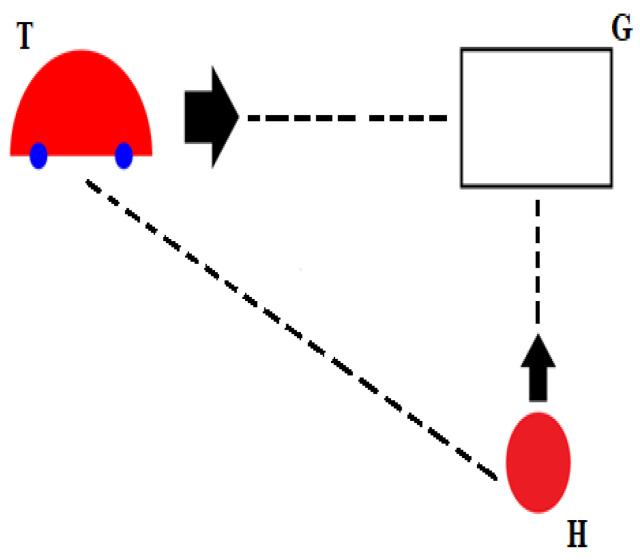
Graphical representation of the experimental task presented on a large touchscreen, where participants had to move up the red dot with the fingertip of their right dominant hand (H) and intercept the horizontally moving target car (T) at the rectangular goal location (G). The car was moving from left to right on the screen towards the interception area under three randomly presented constant accelerations: 0.12 m/s² (slow), 0.33 m/s² (medium), and 0.58 m/s² (fast). The target car (T) was moving towards goal location (G) while the hand (H) was also moving towards G to intercept the moving car.

Psychology Software Tools E-Prime^®^ 2.0 was used to display the experimental stimuli on a Microsoft Surface Hub 1.0 touchscreen 1.17 m by 2.20 m, with a resolution of 2160 × 3840 pixels, operated by an HP Windows computer. The participants were half-standing while leaning their bottom against the edge of a chair as if on a high stool at approximately 70 cm distance from the screen, so they would be able to comfortably reach the touchscreen, on which the target car T, goal location G, and movement dot H were projected. Participants had to vertically move their finger on the touchscreen to intercept the horizontally moving target car at the predetermined catching location G. The size of the car was 57 mm by 35 mm, radius of the movement dot was 18 mm, and the size of the catching location was 59 mm by 42 mm, as displayed in Figure 3. At the start of each trial, the distance between car and the rectangular goal location was 688 mm, while the distance from the dot to the catching location was 660 mm. The car moved under three different constant accelerations that were randomly generated but never repeated in a row: 0.12 m/s² (slow), 0.33 m/s² (medium), or 0.58 m/s² (fast). Obtained car motion and finger movement coordinates were recorded in separate files for further analysis.

### 2.3. Brain Data Acquisition

EEG activity was recorded with a 256-channel Geodesic Sensor Net (GSN) 200 [44] which was evenly distributed over the scalp. The net was connected to a high-input amplifier to ensure that the signals would reach maximum impedance set to 50 kΩ, as recommended for optimal signal-to-noise ratio [45]. EEG signals were recorded with Net Station software at 500 Hz on a Macintosh computer, with applied online low-pass filter (200 Hz) and high-pass filter (0.1 Hz).

Different computers that were working in tandem coordinated the experimental process. HP computer with Windows 10 operating system that was built into the Microsoft Surface Hub 1.0 displayed the experimental stimuli with the help of custom-written Python 3 software. For later offline analyses, the software wrote away additional data and information in separate .txt files, such as car motion and finger movement trajectory coordinates, trial speed condition, and information about whether the interception resulted in a hit or miss. Another HP computer with Windows XP operating system was responsible for E-Prime 2.0 Software and the coordination of the HP Windows 10 computer and Macintosh computer, so that all computers were collecting data simultaneously.

### 2.4. Procedure

Participants arrived at the laboratory, received verbal information and instructions about the study, and signed the informed consent form. They were instructed that there would be a horizontally moving car from left to right on the screen and that they were to intercept it at the designated target location by moving up a red dot with their finger vertically, until they would collide at the target location. Participants were asked not to move their body or head unnecessarily, look straight ahead, and only move their right arm and eyes to complete the task.

**Figure 4:**
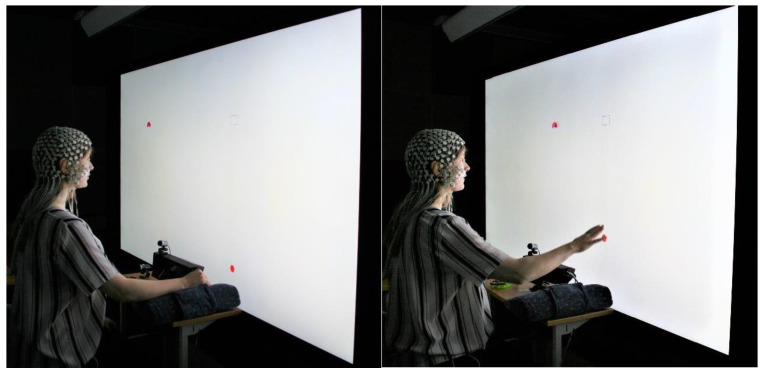
Experimental set up and procedure. While wearing a 256-channel Geodesic Sensor Net, participants sat in front of a large Microsoft SurfaceHub 1.0 4k touchscreen and were asked to move a red dot upwards with the index finger of their right dominant hand to the designated catching location to intercept a horizontally moving car that approached under three different accelerations.

After the experimental procedure instructions, the participant’s head diameter was measured for the appropriate EEG net selection. The chosen net was soaked in a saline electrolyte solution, partially dried with a towel, and placed on the participant’s head. After that, the participant was taken to the experimental room with the touchscreen (Figure 4). Participants were asked to sit on the edge of the chair so that they would be comfortable in reaching the touch screen and could move the arm freely. The EEG net was then connected to the amplifier.

Participants were asked to remove any electronic devices that could disturb the EEG signal. All the assistants moved to the control room behind a glass window, leaving the participant alone in the experimental room, and the impedance of electrodes was checked. If necessary, the impedance was corrected by either adjusting the position of the electrodes on the head or adding extra saline electrolyte solution.

At the start of the experiment, participants performed six practice trials, two at each speed, to familiarize themselves with the experimental set-up and procedure. Every trial was defined as either hit or miss. The car started moving when the participants touched the dot on the screen with their right index finger. Between trials, participants were allowed to rest their arm on a small pillow that was placed on the chair for comfort.

Once the practice trials were completed, the participants were informed that the experiment would begin and that they should continue the experiment the same way. The experiment consisted of a total of 75 trials, which included a randomized order of 25 blocks that each included three trials of each speed condition. In this way, all trials were randomized but it also made sure that a condition would appear no more than twice in a row. Each experimental session took approximately 8 min, and only trials that were marked as hits were used in the data analyses. The average number of accepted hits were 18.1 for slow, 19.8 for medium, and 18.2 for fast speed. The number of bad electrodes that were excluded from the EEG data never exceeded 10% of the total count of 256, with the average of 5% bad electrodes for all participants together.

### 2.5. Behavioral Data Acquisition and Analyses

Finger movement (y-screen coordinates) were extracted with Python 3 software at 100 Hz, according to the refreshing rate capabilities of the touchscreen. For comparison with brain data that were acquired at 500 Hz, the finger movement data were linearly interpolated in MATLAB from 100 Hz to 500 Hz by calculating the average distance between two following data points to match the frequencies at the same time. Time-series of car motion (x-screen coordinates) at three different constant accelerations were generated for 500 Hz and 100 Hz to allow for comparison with brain data and finger movement, respectively.

### 2.6. Brain Data Analyses and Artefact Removal

EEG recordings were segmented by Net Station software and transferred to an external server for analysis purposes. Data analyses were performed with the Brain Electrical Source Analysis (BESA 6.0) software. Averaging epoch length was from −300 ms to 500 ms with a baseline definition of −100 ms to 0 ms with respect to stimulus onset. A notch filter was set to 50 Hz for line interference removal. Then, the data were high-pass filtered at 60 Hz and low-pass filtered at 1.6 Hz. Channels were interpolated to the 81 standard electrodes of the 10-10 system for further analysis. Channels that were corrupted by artefacts and epochs from head or body movements were excluded from the analysis or interpolated.

### 2.7. VEP and MRP Peak Analysis, and Tau-Coupling

Brain areas involved in perception during interception of visual targets are the temporal and posterior parietal areas of the dorsal visual pathway [46,47,48]. In particular, the posterior parietal cortex plays a role in the ongoing guidance of interceptive actions [49,50,51]. Following the dorsal pathway, cortical motor areas are activated [52,53,54], playing a role in sensorimotor transformation and motor execution of the interception task [55].

Using EEG, a visual-motion-related N2 component of visual evoked potentials (VEPs) has been found in adults in the occipital and parietal areas [56,57]. The N2 latency peak seems to increase with faster visual motion speeds [58,59]. The P300 component of movement-related potentials (MRPs) in premotor, motor, and parietal areas is shown to be related to reaching [60,61,62,63,64].

All brain data were combined into a grand average. This grand average was used to select electrodes for the recording of the peak latencies of the N2 component for VEP analysis in occipital and parietal areas, and the P300 component for MRP analysis in central areas. An additional variable was made which included successful hits and was used for selecting the electrodes with the highest mean activation. The electrodes with the highest mean activation of the N2 and P3 component in response to “hits” were: POz, PO4, Oz, and O2 for the VEP analysis, and Cz, C2, and FCz for the MRP analysis. The grand average was also used as a reference to identify the individual N2 and P3 components in the individual averages.

Individual data for slow, medium, and fast speed were averaged and combined into standardized 81-electrode configuration of the 10-10 International system. Brain wave latency peaks were selected for each participant for all three speeds. Peaks were selected around 200–350 ms after the car started moving for N200 [65] and 250–500 ms for P300 [66]. For each participant, electrodes showing good-quality latency peaks and distinctive brain waves around N200 and P300 component peaks were selected, keeping as close to the head midline as possible. The data were filtered using Gaussian sigma 3 filter for brainwaves, car motion, and finger movements, before the following τ-couplings were computed: (1) car vs. time, (2) car vs. finger, (3) VEP vs. MRP, (4) VEP vs. car, and (5) MRP vs. finger. For details about the τ-coupling procedure, see [42,67].

## 3. Results

### 3.1. VEP and MRP Responses

**Figure 5:**
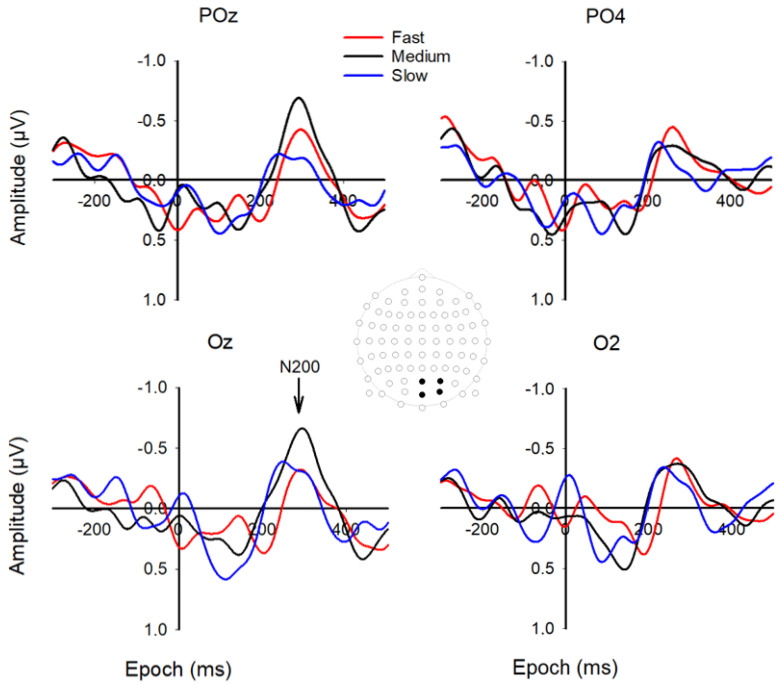
Grand-averaged waveforms of the VEP’s. Epoch is from −300 to 500 ms. The head drawing (nose up) shows scalp locations of the 81 standard channels with the occipitoparietal channels of interest depicted with filled black circles (from top to bottom and left to right): POz, PO4, Oz, and O2. The arrow shows the N2 component of the three speeds at approximately 275 ms.

Four grand averaged channels were selected for VEP analysis, based on their highest mean N200 amplitude, i.e., POz, PO4, Oz, and O2 (see Figure 5), and they were selected as close as possible to the midline of the brain. The average mean VEP N2 latency for the three car speeds slow, medium, and fast was 246 ms (SD = 54), 292 ms (SD = 35), and 286 ms (SD = 44), respectively.

**Figure 6:**
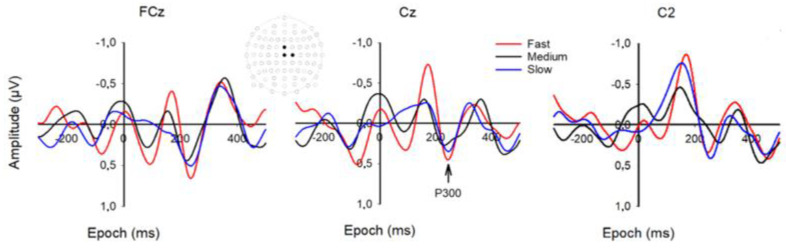
Grand-averaged waveforms of the MRP’s. Epoch is from −300 to 500 ms. The head drawing (nose up) shows scalp locations of the 81 standard channels with the frontocentral channels of interest depicted with filled black circles (from top to bottom and left to right): FCz, Cz, C2. The arrow shows the P3 component of the three speeds at approximately 295 ms.

Three grand averaged channels were selected for MRP analysis, based on the highest mean P300 amplitude, i.e., FCz, FC2, and Cz (see Figure 6). The average mean MRP P3 latency was 295 ms (SD = 87) for slow, 284 ms (SD = 81) for medium, and 297 ms (SD = 107) for fast speed.

### 3.2. Tau-Coupling Results

1. Car vs. Time

**Figure 7:**
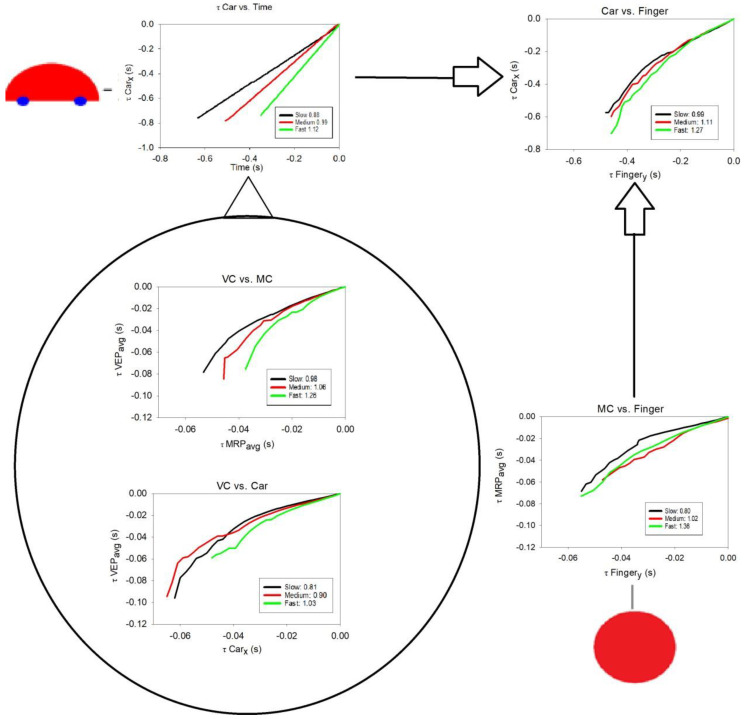
Overall τ-coupling results, depicting five grand averaged τ-coupling graphs overlayed on a task/head model that reflect the ecological scale and neural scale of the interception task. The graphs display the τ-coupling results of (1, **top left**) car vs. time-to-collision, (2, **top right**) car motion vs. finger movement, both representing the ecological scale, and (3, top graph in the head model) visual cortex (VEP) vs. motor cortex (MRP), (4, bottom graph in the head model) visual cortex (VEP) vs. car motion, and (5, **bottom right**) motor cortex (MRP) vs. finger movement, with 3–5 representing the neural scale of the interception task. Each graph displays the average slopes for each τ-coupling constant k for the three different car speeds slow (black), medium (red), and fast (green). For details about the τ-coupling procedure, see [42].

Figure 7 and Table 1 show the results of plotting the τ-values of car motion (in x-screen coordinates) against actual time-to-collision for the three car speeds (constant accelerations). These plots were included in the analyses to indicate the veridical car speed during the task. The resulting coupling constant *k* for each car speed indicates the perceptual variables involved in the interception task at the ecological scale. Strictly speaking, this is not a τ-coupling as such, since it is only the τ-value of the moving car that was plotted against time [22]. The results show that the slope values (*k*) for slow, medium, and fast speed when τ_car_ was plotted against time were 0.88 (SD = 0.06) for slow, 0.99 (SD = 0.09) for medium, and 1.12 (SD = 0.09) for fast moving cars. We will use these benchmark values as reference during the rest of our analyses when considering τ-coupling plots at both the ecological and neural level of analysis.

2. Car vs. Finger: Ecological One-Scale Indirect Comparison with Neural Scale Via *k*

A τ-coupling analysis was conducted between car motion (x-screen coordinates) and finger movements (y-screen coordinates) for the three car speeds (see Figure 7 and Table 1). The average τ-coupling slope values (*k*) for slow, medium, and fast car speed were 0.99 (SD = 0.07), 1.11 (SD = 0.09), and 1.26 (SD = 0.08), respectively. A repeated-measures ANOVA showed the slope values between τ_car_ and τ_finger_ increased significantly with higher car speeds, *F* (2,22) = 52.85, *p* < 0.001. The average percentage τ-coupling was over 95% for all car speeds, and average r^2^ values were generally high and over 0.96, as can be seen in Supplementary Table S1.

3. VC (VEP) vs. MC (MRP): Neural, Same-Scale Indirect Coupling with Ecological Scale via *k*

A τ-coupling analysis was conducted between evoked responses in the motor and visual cortices for the three car speeds, comparing subject averages for MRP and VEP latency peaks (see Figure 7 and Table 1). The average τ-coupling slope values (*k*) between VEP and MRP signals for slow, medium, and fast car speed were 0.98 (SD = 0.14), 1.06 (SD = 0.16), and 1.26 (SD = 0.17), respectively. When τ_VEP_ was plotted against τ_MRP_, a repeated-measures ANOVA showed a significant increase in slope values with higher car speeds, *F* (2,22) = 16.52, *p* < 0.001. The average percentage τ-coupling was over 91% for all car speeds, and average r^2^ values were over 0.98, as can be seen in Supplementary Table S1.

4. VC (VEP) vs. Car: Ecological and Neural, Two-Scale Direct Comparison via *k_res_*

A τ-coupling analysis was conducted between the average visual cortex (VEP) waveforms and car coordinates for the three car speeds. The average τ-coupling slope values (k_res_) for slow, medium, and fast car speed between VEP activity and car motion were 0.81 (SD = 0.09), 0.90 (SD = 0.13), and 1.03 (SD = 0.13), respectively (see Figure 7 and Table 1). A repeated-measures ANOVA showed that when τ_VEP_ was plotted against τ_car_, slope values increased significantly with higher car speeds, *F* (2,22) = 17.17, *p* < 0.001. The average percentage τ-coupling was over 87% for all speeds, and the average r^2^ values were over 0.96, as seen in Table S1.

5. MC (MRP) vs. Finger: Neural and Ecological, Two-Scale Direct Comparison via *k_res_*

A τ-coupling analysis was conducted between average MRP latency peaks and finger coordinates for the three car speeds (see Figure 7 and Table 1). The average τ-coupling slope values (k_res_) for slow, medium, and fast car speed between MRP activity and finger movements were 0.80 (SD = 0.15), 1.02 (SD = 0.32), and 1.36 (SD = 0.39), respectively. A repeated-measures ANOVA showed that when τ_MRP_ was plotted against τ_finger_, slope values increased significantly with higher car speeds, *F* (2,22) = 7.32, *p* < 0.01. The average percentage τ-coupling was over 86% for all speeds, and the average r^2^ values were over 0.95 (see Table S1).

## 4. Discussion

Formally, the theory of τ-coupling is described by [23] as τ_HG_ = *k*τ_TG_ (see Equation (1)). In this paper, we proposed to consider this description to be a particular instantiation of the more general ecological resonance equation χ = *k*ψ (see Figure 2). Thus, we regarded τ-coupling to be formally equivalent to ecological resonance (via *k* and *k_res_*) with the specification that both the ecological scale and the neural scale are present in the description. We presented a combination of both descriptions, i.e., τ-coupling via *k* and *k_res_*, to illustrate how ecological resonance can be described involving both same-scale and two scales of analyses. In the context of τ-coupling, the presence of τ in neural activity can be seen as strong evidence for ecological resonance and, more generally, for the use of ecological information in the control of behavior. τ is relatively simple in mathematical terms, but it is not an obvious property of the optical flow. It is the inverse of the relative rate of dilation of a closed contour in the visual field, which is less intuitively straightforward than speed or intensity. It is not obvious at all that we should find τ-coupling at the neural scale, and yet we did. With this in mind, we now discuss same-scale and two-scale τ-coupling analyses using *k* and *k_res_* in the context of ecological resonance.

### 4.1. Car vs. Time

First, we discuss the relationship between the movement of the target car and time-to-collision. This is not a τ-coupling analysis as such, since it is only the τ-value of the moving car that was plotted against the actual time-to-collision. Lee [22] described how, in the case of braking a car, the visual variable τ, which specifies the time-to-collision if the closing velocity were maintained, and its time derivative τ-dot could be used by the perceiver in determining the type of course he or she is on and, hence, the action which needs to be taken depending on different ranges of k (see Introduction). Figure 7 and Table 1 showed the results of plotting the τ-value of the target car against actual time-to-collision for the three car speeds. The resulting coupling constant k for each car speed reflects the perceptual variables that are involved in the interception task at the ecological scale. The results showed that the *k* values for slow, medium, and fast speed between τ_car_ and time-to-collision increased from 0.88 for slow, to 0.99 for medium, and to 1.12 for fast-moving target cars. For slow and medium car speeds, *k* was kept below 1, indicating hard contact with the goal location, whereas for fast speed, *k* was greater than 1, indicating accelerating contact with the goal location. These benchmark values were used as reference during the rest of our analyses when considering τ-coupling plots at both the ecological and neural levels of analysis.

### 4.2. Indirect Same-Scale τ-Coupling Analysis via k

#### 4.2.1. At the Ecological Scale: Car vs. Finger

The average τ-coupling slope values (*k*) for the same-scale analysis at the ecological scale between car motion and finger movements increased linearly from 0.99 to 1.11 to 1.26 with increasing car speed. Comparing these slope results with the benchmark values from Car vs. Time, it appears that when finger movements were involved, average *k* values were overall approximately 10% higher, revealing a general tendency in the participants to intercept the moving car at the goal location with a higher acceleration than necessary. In addition, participants differentiated well between the three car speeds when intercepting the moving car with their finger. Our results are consistent with empirical evidence for τ-coupling on a similar interception task [1].

#### 4.2.2. At the Neural Scale: VC (VEP) vs. MC (MRP)

The average τ-coupling slope values (*k*) for the same-scale analysis at the neural scale between VEP and MRP signals increased linearly from 0.98 to 1.27 from slow to fast car speed. When comparing these slope results with the benchmark coupling values from the section on Car vs. Time, we can see that average *k* values were again approximately 10% higher, yet they were very similar to the results discussed in the previous section. The results showed again a clear tendency in all participants to differentiate well between the three car speeds as far as brain activity was concerned. Therefore, slope (*k*) results seem to be independent of the scale of analysis, i.e., ecological or neural.

The question is: How can these two same-scale τ-coupling analyses at the two different scales contribute to the understanding of the concept of resonance? First, the ecological scale seems to be reflected in the neural scale. This entails that the descriptions developed at each of the scales via τ-coupling are not in conflict, but reciprocal. Moreover, it is not clear that there is any need for the reduction of one to the other. They can be regarded as two complementary descriptions of the same phenomenon about the same perception-action system. The question is not whether one scale can be reduced to the other, but how we can understand the relationship between the scales in a coherent manner. A resonance-based framework offers a clear way to tackle this question [27,41].

Second, the dynamics at the ecological and neural scales of analysis are connected by task-relevant information in a fully operative fashion. The information available at the ecological scale has been thoroughly studied in ecological psychology [16,68,69], and resonance is a well-known physical process. Given that, the framework fits well with one important tenet of ecological psychology, namely that neural systems are best understood in terms of the way their dynamics are constrained by the information generated in organism–environment interactions. In this sense, a resonance-based framework provides a way to generate and test hypotheses regarding the connection of the two relevant scales of analysis. Our study is an example of such a hypothesis.

### 4.3. Direct Two-Scale τ-Coupling Analysis via k_res_

In the direct τ-couplings, variables at the ecological scale are directly coupled to variables at the neural scale resulting in a direct measure of resonance via the coupling constant *k_res_*. Time scales are typically rather different in these kinds of direct couplings. Where variables at the ecological scale typically are measured in seconds, they are measured in milliseconds at the neural scale. However, the τ-coupling procedures used are robust enough to handle these differences and allow us to show underlying kinematic profiles from the ecological level when plotted directly on the scale of the neural level. This is possible because whereas time-to-contact, τ(t), is measured in seconds, its derivative τ-dot is an elementary dimensionless quantity that can be used in τ-coupling procedures when correlating variables and different time scales of varying magnitude resulting in the informative coupling constant *k_res_*.

#### 4.3.1. VC (VEP) vs. Car

A τ-coupling analysis was conducted between the average visual cortex (VEP) latency peaks and car motion coordinates for each of the three car speeds. Average τ-coupling slope values (*k_res_*) increased significantly from 0.81, to 0.90, and to 1.03 for slow, medium, and fast car speed, respectively. When comparing these slope results with the benchmark coupling values from the section on Car vs. Time, it turns out that average *k_res_* values were overall approximately 10% lower, revealing a general tendency for participants to deal with motion gap closures at the neural level in a softer manner. At the same time, participants differentiated well between the three car speeds in their VEP activity, showing more forceful neural gap closures with increasing car speeds.

#### 4.3.2. MC (MRP) vs. Finger

Average τ-coupling slope values (*k_res_*) for the two-scale analysis between the average MRP activity and finger movements increased significantly from 0.80, to 1.02, and to 1.36 for slow, medium, and fast car speed, respectively. This time, average *k_res_* values were more varying and with higher standard deviations compared to benchmark coupling values for Car vs. Time. For slow car speed, coupling values were approximately 10% lower, whereas for high car speed they were approximately 20% higher. For medium speed, coupling values were roughly the same. These results suggest that performance was more varied whenever finger movements were involved in the τ-coupling. Again, participants differentiated well between the three car speeds in their MRP activity, showing increasingly more forceful neural gap closures with faster car speeds.

These findings are compatible with our earlier τ-coupling findings on infants’ interceptive actions when catching moving toys [11,70]. Humans are relatively insensitive to perceiving acceleration [71]. When determining the time-to-collision of a moving object, we appear to assume the object is approaching under constant velocity, resulting in an under-/overestimation of the actual time it will take the object to reach us if it were accelerating/decelerating, respectively. The present findings suggest that participants dealt with motion gap closures at the neural level as if the car was approaching at a constant speed and compensated for the resulting underestimation of the remaining time-to-collision by producing finger movements that collided harder with the car in the goal location to successfully complete the interceptive timing task.

## 5. Conclusions

When τ-coupling between two different scales of analysis, we showed how the concept of τ-coupling can contribute to the understanding of the concept of resonance using the coupling constant *k_res_*. By considering *k_res_* directly, we were able to determine how variables at the ecological scale relate to variables at the neural scale and, as such, provide us with a direct measure characterizing the degree of resonance between them, as well as testifying that the temporal structure of controlling the movement of effectors across gaps to destinations is sustained during the neural control of these actions. The idea that relevant information generated at the scale of the dynamics of the organism–environment interactions constrains the dynamics of the neural system is not just a speculative proposal. On the contrary, there are also other empirical results that describe events in which this kind of or similar information is constraining neural dynamics of different systems [42,67,72,73,74,75].

Within the field of ecological psychology, it has long been considered adequate to investigate informational variables at the interactive level to describe the relationship between the organism and the environment. However, several researchers have recently started asking questions about the ways in which the particulars of the organism’s nervous system are involved, e.g., [76]. In the present paper, we have argued that a complete description of behavior requires inclusion of neural aspects. To this end, we presented τ-coupling as a valuable analysis tool to combine variables at the different scales of operation when describing interceptive actions. The results were subsequently used to show how ecological resonance can be applied as a plausible concept to understand such actions. In our view, these concepts provide a promising way to investigate behavior and brain from an ecological neuroscience perspective that can be applied across the lifespan to study typically and atypically developing neural systems.

## Figures and Tables

**Table 1:** Grand average slope values of τ-coupling constant *k* and *k_res_* for the three car speeds slow, medium, and fast and their standard deviations.

Type of τ-Coupling	Resonance Scale	Slow (SD)	Medium (SD)	Fast (SD)
1. Car vs. Time (benchmark values)	Ecological scale (not a τ-coupling as such)	0.88 (0.06)	0.99 (0.09)	1.12 (0.09)
2. Car vs. Finger	Ecological, same-scale indirect coupling comparison with neural scale via *k*	0.99 (0.07)	1.11 (0.09)	1.26 (0.08)
3. VC (VEP) vs. MC (MRP)	Neural, same-scale indirect coupling comparison with ecological scale via *k*	0.98 (0.14)	1.06 (0.16)	1.27 (0.17)
4. VC (VEP) vs. Car	Ecological and neural, two-scale direct coupling comparison via *k*_res_	0.81 (0.09)	0.90 (0.13)	1.03 (0.13)
5. MC (MRP) vs. Finger	Neural and ecological, two-scale direct comparison via *k*_res_	0.80 (0.15)	1.02 (0.32)	1.36 (0.39) *

* The five most relevant τ-coupling events describe the same-scale activity at the ecological scale and the neural scale (1, 2, 3), and the two-scale combinations of the ecological scale and the neural scale (4, 5). These are the τ-couplings of: (1) car motion vs. time-to-collision which describes the veridical perceptual information at the ecological scale, (2) car motion vs. finger movement, (3) visual cortex (VEP) vs. motor cortex (MRP), (4) car motion vs. visual cortex (VEP), and (5) motor cortex (MRP) vs. finger movement. The arrows between 2 and 3 indicate that k-values can be indirectly compared across scales for each car speed separately.

## Data Availability

Data supporting this manuscript will be made available on reasonable request. The manuscript has summary data included as electronic supplement.

## Supplementary Materials

The following supporting information can be downloaded at: https://www.mdpi.com/article/10.3390/brainsci12121737/s1, Table S1: Detailed overview of grand averaged τ-coupling results. * Tau-coupling relations are depicted between brain activity in the visual and motor cortices, finger movement, and car motion. Each coupling includes either the coupling between two variables or with the differentiation between MRP and VEP when the coupling includes brain activity, to see how the different brain regions respond to the same information. Each coupling includes grand average slopes (τ-coupling constant *k*), the strength of the τ-coupling indicated by r^2^, and finally the percentage of τ-coupling during the entire trial.
