# Supplementary material for: Neural Aspects of Prospective Control through Resonating Taus in an Interceptive Timing Task
Source: Brain Sci. 2022 Dec 19;12(12):1737. doi: 10.3390/brainsci12121737 (PMC9776417; doi:10.3390/brainsci12121737)
Supplement: Supplementary file 1 [file brainsci-12-01737-s001.zip › brainsci-2074894-supplementary.pdf]

Supplementary Table S1. Detailed overview of grand averaged τ-coupling results.

**Brain activity**		**Finger movement**					
		**MRP**			**VEP**		
		Slow (SD)	*Medium (SD)*	*Fast (SD)*	*Slow (SD)*	*Medium (SD)*	*Fast (SD)*
	Slope	0.80 (0.15)	1.02 (0.32)	1.36 (0.39)	0.87 (0.09)	0.96 (0.45)	1.24 (0.42)
	r^2	0.96 (0.01)	0.96 (0.01)	0.96 (0.01)	0.96 (0.01)	0.97 (0.02)	0.97 (0.01)
	% Total	86 (10.70)	89 (10.23)	89 (7.57)	92 (9.87)	95 (7.71)	87 (9.50)
**Brain activity**		**Car movement**					
		**MRP**			**VEP**		
		Slow (SD)	*Medium (SD)*	*Fast (SD)*	*Slow (SD)*	*Medium (SD)*	*Fast (SD)*
	Slope	0.85 (0.06)	0.97 (0.12)	1.07 (0.07)	0.81 (0.09)	0.90 (0.13)	1.03 (0.13)
	r^2	0.96 (0.01)	0.96 (0.01)	0.96 (0.01)	0.96 (0.01)	0.96 (0.01)	0.96 (0.01)
	% Total	83 (9.65)	85 (9.42)	80 (6.91)	84 (9.74)	90 (9.93)	85 (9.90)
**Brain activity (MRP)**		**Brain activity (VEP)**					
		Slow (SD)		*Medium (SD)*		*Fast (SD)*	
	Slope	0.98 (0.14)		1.06 (0.16)		1.27 (0.17)	
	r^2	0.98 (0.02)		0.98 (0.02)		0.98 (0.02)	
	% Total	94 (9.17)		96 (8.43)		91 (10.90)	
**Car motion***		**Finger movement**					
		Slow (SD)		*Medium (SD)*		*Fast (SD)*	
	Slope	0.99 (0.07)		1.11 (0.09)		1.26 (0.08)	
	r^2	0.96 (0.01)		0.96 (0.01)		0.97 (0.01)	
	% Total	95 (6.02)		97 (5.38)		98 (4.71)	

* Tau-coupling relations are depicted between brain activity in the visual and motor cortex, finger movement, and car motion. Each coupling includes either the coupling between two variables or with the differentiation between MRP and VEP when the coupling includes brain activity, to see how the different brain regions respond to the same information. Each coupling includes grand average slopes (τ-coupling constant *k*), the strength of the τ-coupling indicated by r^2, and finally the percentage of τ-coupling during the entire trial.
